# Impact of chronic kidney disease on clinical outcomes in patients with Stage B progressive aortic regurgitation (mild to moderate and moderate grades)

**DOI:** 10.1002/clc.23792

**Published:** 2022-02-16

**Authors:** Ji‐won Hwang, Dong‐Gil Kim, Hakju Kim, Jae‐Jin Kwak, Sung Woo Cho, Da Mi Bae, Yoon Cheol Shin, Joon Hyung Doh, Sung Uk Kwon, June Namgung, Sung Yun Lee

**Affiliations:** ^1^ Department of Internal Medicine, Division of Cardiology Inje University College of Medicine, Ilsan Paik Hospital Goyang Korea; ^2^ Department of Thoracic and Cardiovascular Surgery Inje University College of Medicine, Ilsan Paik Hospital Goyang Korea

**Keywords:** chronic kidney disease, heart failure, progressive aortic regurgitation

## Abstract

**Background:**

Chronic kidney disease (CKD) is a significant comorbidity in patients with heart failure and valvular heart disease. Renal impairment is not well evaluated in the patients with Stage B progressive aortic regurgitation (AR) (mild to moderate and moderate grades in this study), for estimating outcome.

**Hypothesis:**

We sought to investigate the prognostic factor, especially CKD, in the patients with progressive AR.

**Methods:**

We enrolled 262 patients with Stage B progressive AR and preserved left ventricular systolic function (ejection fraction ≥ 50%). Based on the presence of CKD, the patients were divided into CKD (*n* = 70) and non‐CKD (*n* = 192) groups, which CKD was defined as estimated glomerular filtration rate < 60 ml/min/1.73 m^2^. The primary outcome was major adverse cardiac events (MACEs), including cardiac death, myocardial infarction, hospitalization for heart failure, and aortic valve replacement.

**Results:**

The median follow‐up duration was 41.5 (interquartile range: 16.2–71.7) months. Between groups, the CKD patients were older; they had a higher pulse pressure and higher incidence of hypertension, diabetes mellitus, dyslipidemia, cerebrovascular accident, and atrial fibrillation. Compared to the non‐CKD group, the CKD group had lower *e*ʹ velocity (4.36 ± 2.21 vs. 5.20 ± 2.30 cm/s, *p* = .009), higher right ventricular systolic pressure (38.02 ± 15.79 vs. 33.86 ± 11.77 mmHg, *p* = .047). The CKD group was associated with increased risk of MACEs (41.4% vs. 22.4%; unadjusted hazard ratio [HR]: 1.78, 95% confidence interval [CI]: 1.11–2.85, *p* = .017). In multivariate Cox regression analyses, the risk of MACEs was significantly different between groups (adjusted HR: 1.71, 95% CI: 1.11–2.62, *p* = .015); furthermore, the risk of hospitalization for heart failure (10.0% vs. 2.6%; adjusted HR: 2.30, 95% CI: 1.16–4.55, *p* = .017) was significantly higher in the CKD group than in the non‐CKD group.

**Conclusions:**

In patients with Stage B progressive AR, CKD is an independent prognostic factor for clinical outcomes (composite clinical outcome, hospitalization for heart failure).

## Highlights

- Chronic kidney disease is a known comorbidity for valvular heart disease.
- We investigated that chronic kidney disease was an independent prognostic factor for clinical outcomes (composite clinical outcome and hospitalization for heart failure) in patients with Stage B progressive aortic regurgitation.
- Although Stage B progressive aortic regurgitation should be carefully monitored in all patients by guideline, special attention should be paid to patients with pre‐existing chronic kidney disease.

## 1 INTRODUCTION

Aortic regurgitation (AR) is one of the causes of left‐sided structural heart disease. The natural history of AR is well defined; it has a gradually progressive clinical course with effects on the left ventricle, including eccentric hypertrophy, fibrosis, and ultimately, cardiac failure.^1^ In addition, many studies have been conducted on factors that influence AR progression.

Approximately 6% of the patients with AR develop symptoms of left ventricular (LV) systolic dysfunction each year; moreover, the mortality rate is about 10% per year in the presence of LV systolic dysfunction.^1^ However, for Stage B progressive AR (mild to moderate and moderate grades of AR),^2^ relevant studies are scarce. Further, the clinical features progressing to heart failure or cardiovascular outcome have not been elucidated. In the context of mild and moderate AR, there is only very limited data on the long‐term progression and follow‐up.^3^, ^4^ The current guidelines for surveillance echocardiography recommends appropriate periodic testing, every 2–3 years, for monitoring Stage B progressive AR patients.^5^, ^6^

Chronic kidney disease (CKD) is a significant comorbidity for most major cardiovascular diseases^7^, ^8^; it is associated with significant mortality and morbidity in patients with valvular heart disease or those undergoing valvular procedures, especially for calcified valvular disease (such as aortic stenosis or mitral stenosis).^9^, ^10^, ^11^

There is not much clinical evidence on the prognostic factors for Stage B progressive AR, and little information is available on the effects of CKD in these patients. This study aimed to evaluate the prognostic factors, especially the impact of CKD, in patients with Stage B progressive AR.

## 2 METHODS

### 2.1 Study population and clinical characteristics

In this retrospective cohort study, we retrieved data of patients with a mild to moderate or higher grade of valvular heart disease from the echocardiographic registry of our institution, between 2010 and 2019.

First, we searched the registry to identify patients with AR (*n* = 645) and reviewed their medical records. Next, we selected patients with a medical record of follow‐up and excluded those with the following conditions: severe grade of AR (Stages C and D)^5^; concomitant with ≥moderate grade of aortic stenosis, mitral stenosis, and mitral regurgitation; previous surgery involving the aortic valve and mitral valve; congenital heart disease; hypertrophic, dilated, or ischemic cardiomyopathy; and end‐stage renal disease requiring hemodialysis. Finally, a total of 262 patients who had Stage B progressive AR (mild to moderate and moderate grades of AR) and preserved LV systolic function (ejection fraction ≥ 50%) were enrolled.

For all patients, the data, including clinical characteristics and laboratory findings, were carefully reviewed by a single cardiologist. The study protocol was approved by the institutional review board of our institution, and the need for informed consent was waived.

We divided our study cohort into two groups based on their renal function: the CKD group and the non‐CKD group. The CKD was defined as estimated glomerular filtration rate (eGFR) less than 60 ml/min/1.73 m^2^. The values of laboratory findings (such as creatinine) were collected between 1 month before and after the echocardiography was performed. For eGFR values, the Chronic Kidney Disease Epidemiology Collaboration (CKD‐EPI) equation was used.

### 2.2 Echocardiographic measurement

Commercially available ultrasound equipment was used for echocardiographic studies. Conventional two‐dimensional echocardiography was performed, and echocardiographic parameters were obtained following the American Society of Echocardiography guidelines. LV end‐diastolic and end‐systolic volumes were measured using apical two‐ and four‐chamber views, and the ejection fraction was calculated using Simpson's rule.^12^ Left atrial volume was calculated using the modified biplane area‐length formula and was indexed to the patient's body surface area for the left atrial volume index. Early diastolic mitral inflow velocity (*E* velocity) was measured using the pulsed‐wave Doppler method by placing the sample volume at the tip of the leaflets of the mitral valve. Tissue Doppler‐derived early‐diastolic mitral annular velocity (*e*ʹ velocity) was measured at the septal corner of the mitral annulus in the apical four‐chamber view. In addition, deceleration time was measured for the early transmitral flow velocity. *E*/*e*ʹ ratio was used to estimate the LV filling pressure.^12^

For assessment of AR severity, an integrated echocardiography examination, including visual parameters, the AR jet width at the aortic valve, the jet height in the LV outflow tract, the strength of the AR continuous wave signal, and the pressure half time, was performed. The presence of pan‐diastolic flow reversal in the proximal descending aorta was considered to indicate severe AR.^5^, ^6^ AR severity was derived using a multiparametric approach and classified according to the guidelines. Echocardiography data were reanalyzed by an experienced imaging cardiologist for consistency.

### 2.3 Clinical outcomes

The date of the patient's baseline echocardiography was considered the beginning of the observation period. All enrolled patients were followed up via a chart review, and the date of last follow‐up or death was recorded. The patients, whose end point was unknown, were treated as follow‐up loss patients using statistics. All outcome events were adjudicated by investigators.

The primary outcome was a composite clinical outcome—major adverse cardiac events (MACEs), including cardiac death, myocardial infarction, hospitalization for heart failure, and aortic valve replacement. The secondary outcome was hospitalization for heart failure.

### 2.4 Statistical analysis

Continuous variables were compared using Student's *t*‐test and presented as the mean with standard deviation or median with interquartile range (IQR). Categorical data were tested using Fisher's exact test or *χ* ^2^ test, as considered appropriate.

Independent prognostic factors for clinical outcomes were analyzed using Cox proportional hazards regression models. Results were presented as hazard ratios (HRs) with corresponding 95% confidence intervals (CIs). Variables from univariate analyses with *p* < .20 or clinically meaningful parameters were then entered into a multivariate Cox regression model. Demographic parameters such as age and sex were necessarily included in clinically meaningful parameters, and hypertension (HTN), diabetes mellitus (DM), and dyslipidemia were also applicable in these. Eventually, adjusted HRs were compared using multivariate Cox proportional hazards stepwise regression model based on age, sex, pulse pressure, HTN, DM, hyperlipidemia, cerebrovascular accident, atrial fibrillation, and *e*ʹ velocity.

In addition, a Kaplan–Meier analysis was used to describe the event‐free survival, and the log‐rank test was used to analyze the differences between the groups with and without CKD.

All tests were two‐tailed, and values of *p* < .05 were considered statistically significant. All statistical analyses were performed using Statistical Package for the Social Sciences (IBM SPSS Statistics for Windows, Version 23.0; IBM Corp.).

## 3 RESULTS

### 3.1 Baseline characteristics between the group with and without CKD

Our final cohort consisted of 262 patients, divided into two groups: CKD (*n* = 70) and non‐CKD (*n* = 192). Table 1 summarizes the baseline clinical characteristics and laboratory findings of all patients. In comparison, the patients with CKD were older (75.63 ± 9.18 vs. 70.45 ± 12.14 years). A higher proportion of AR patients with CKD presented with high pulse pressure, HTN, DM, dyslipidemia, cerebrovascular accident, and atrial fibrillation.

**Table 1:** Baseline clinical characteristics of study population divided into the groups with and without chronic kidney disease

	Non‐CKD group (*N* = 192)	CKD group (*N* = 70)	*p *Value
Sex (male)	86 (44.8%)	38 (54.3%)	.21
Age (years)	70.45 ± 12.14	75.63 ± 9.18	<.001
Systolic blood pressure (mmHg)	128.80 ± 14.43	131.60 ± 13.37	.16
Diastolic blood pressure (mmHg)	68.31 ± 11.00	66.19 ± 9.78	.16
Pulse pressure (mmHg)	60.49 ± 13.75	65.41 ± 12.47	.009
Heart rate (bpm)	74.03 ± 12.97	73.03 ± 14.61	.59
Height (cm)	143.03 ± 49.60	153.09 ± 33.87	.06
Weight (kg)	50.51 ± 20.80	54.73 ± 15.63	.12
Body mass index (kg/m^2^)	19.71 ± 7.62	21.33 ± 5.55	.06
NYHA class ≥ II	54 (28.1%)	43 (61.4%)	<.001
Past history			
Hypertension	157 (81.8%)	67 (95.7%)	.005
Diabetes mellitus	29 (15.1%)	20 (28.6%)	.019
Dyslipidemia	114 (59.4%)	57 (81.4%)	.001
Dilated cardiomyopathy	4 (2.1%)	1 (1.4%)	1.00
Coronary artery disease	44 (22.9%)	24 (34.3%)	.08
Cerebrovascular accident	27 (14.1%)	19 (27.1%)	.017
Atrial fibrillation	38 (19.8%)	26 (37.1%)	.006
Abnormal thyroid function	7 (3.6%)	5 (7.1%)	.31
History of cancer	35 (18.2%)	15 (21.4%)	.60
Liver cirrhosis	6 (3.1%)	1 (1.4%)	.68
Bronchial airway disease	27 (14.1%)	15 (21.4%)	.18
Interstitial lung disease	4 (2.1%)	0	.58
Social history			
Alcohol	17 (8.9%)	8 (11.4%)	.64
Current smoker	29 (15.1%)	9 (12.9%)	.84
Ex‐smoker	36 (18.8%)	17 (24.3%)	.39
Status of aortic valve and aorta			
Rheumatic valve disease	2 (1.0%)	3 (4.3%)	.12
Bicuspid aortic valve	3 (1.6%)	0	.57
Quadricuspid aortic valve	3 (1.6%)	1 (1.4%)	1.00
Cusp prolapse	4 (2.1%)	0	.58
Failure of cusp coaptation	17 (8.9%)	4 (5.7%)	.61
Aneurysm of ascending aorta	5 (2.6%)	0	.33
Dilatation of root or ascending aorta	67 (34.9%)	32 (45.7%)	.12
Medication			
Antiplatelet agent	84 (43.8%)	41 (58.6%)	.037
Anticoagulation agent	35 (18.2%)	16 (22.9%)	.48
Digoxin	11 (5.7%)	5 (7.15)	.77
Beta blocker	59 (30.7%)	35 (50.0%)	.006
Calcium channel blocker	77 (40.1%)	42 (60.0%)	.005
Diuretics	52 (2.1%)	31 (44.3%)	.011
Spironolactone	15 (7.8%)	11 (15.7%)	.07
Renin angiotensin system blockade	113 (58.9%)	34 (48.6%)	.16
Statin	114 (59.4%)	54 (77.1%)	.009
Vasodilator	47 (24.5%)	25 (35.7%)	.09
Laboratory finding			
NT‐proBNP (pg/ml)	1095.22 ± 4151.90 0 (0–111.13)	6442.06 ± 13 572.32 0 (0–6596.25)	<.001
Hemoglobin (g/dl)	12.71 ± 2.36	12.11 ± 1.81	.06
Total cholesterol (mg/dl)	141.22 ± 57.04	129.86 ± 64.44	.20
Low density lipoprotein (mg/dl)	83.32 ± 41.67	79.51 ± 44.41	.52
High density lipoprotein (mg/dl)	45.52 ± 22.64	38.91 ± 22.97	.038
Triglyceride (mg/dl)	98.52 ± 88.01	87.11 ± 53.18	.31

*Note*: Data are presented as number of patients (percent) or average ± standard deviation or median with interquartile range (median [IQR]).

Abbreviations: CKD, chronic kidney disease; NT‐proBNP, N‐terminal probrain natriuretic peptide; NYHA, New York Heart Association.

In addition, the CKD group showed higher levels of N‐terminal probrain natriuretic peptide (NT‐proBNP) than the non‐CKD group (6442.06 ± 13 572.32 vs. 1095.22 ± 4151.90 pg/ml, *p* < .001). NT‐proBNP level in blood is affected by creatinine level, that is, renal function. Since the two groups were divided according to the presence or absence of CKD, we considered that the NT‐proBNP level was inevitably statistically different between two groups.

### 3.2 Echocardiographic parameters

The parameters obtained by echocardiography are shown in Table 2. Of the 262 patients, 140 patients had a mild‐moderate grade of AR, and 122 patients had a moderate grade of AR; however, there was no statistically significant difference in the grades of AR between the groups with and without CKD.

**Table 2:** Echocardiographic parameters of study population

	Non‐CKD group (*N* = 192)	CKD group (*N* = 70)	*p* Value
Grade of aortic regurgitation			.21
Mild to moderate	98 (51.0%)	42 (60.0%)	
Moderate	94 (49.0%)	28 (40.0%)	
Pressure half time (ms)	216.02 ± 218.92	231.66 ± 214.46	.61
Jet width in LVOT (%)	35.15 ± 13.26	35.30 ± 12.72	.93
Left ventricular ejection fraction (%)	65.15 ± 6.70	64.86 ± 7.09	.76
Left ventricle end‐diastolic dimension (mm)	51.18 ± 5.78	52.02 ± 4.52	.27
Left ventricle end‐systolic dimension (mm)	31.35 ± 5.72	32.13 ± 4.96	.31
Size of annulus (mm)	7.46 ± 10.32	9.91 ± 11.08	.11
Size of sinus of Valsalva (mm)	14.64 ± 20.30	18.39 ± 20.51	.19
Size of sinotubular junction (mm)	11.77 ± 16.65	15.79 ± 17.61	.10
*E* velocity (m/s)	0.59 ± 0.27	0.59 ± 0.34	.91
*A* velocity (m/s)	0.68 ± 0.36	0.77 ± 0.41	.12
*E*/*A* ratio	0.65 ± 0.46	0.66 ± 0.47	.90
Deceleration time (msec)	225.78 ± 87.26	222.70 ± 119.28	.84
*e*ʹ Velocity (cm/s)	5.20 ± 2.30	4.36 ± 2.21	.009
*a*ʹ Velocity (cm/s)	7.25 ± 3.71	6.89 ± 3.88	.49
E/*e*ʹ ratio	11.51 ± 8.00	12.39 ± 7.84	.43
Left atrium volume index (ml/m^2^)	27.69 ± 23.99	28.40 ± 34.47	.87
Right ventricular systolic pressure (mmHg)	33.86 ± 11.77	38.02 ± 15.79	.047
Significant tricuspid regurgitation	24 (12.5%)	9 (12.9%)	1.00

*Note*: Data are presented as number of patients (percent) or average ± standard deviation.

Abbreviations: CKD, chronic kidney disease; LVOT; left ventricular outflow tract.

The *e*ʹ velocity (4.36 ± 2.21 vs. 5.20 ± 2.30 cm/s, *p* = .009) was significantly higher in patients without CKD.

### 3.3 Cox proportional analysis and survival analysis for clinical outcomes

**Figure 1:**
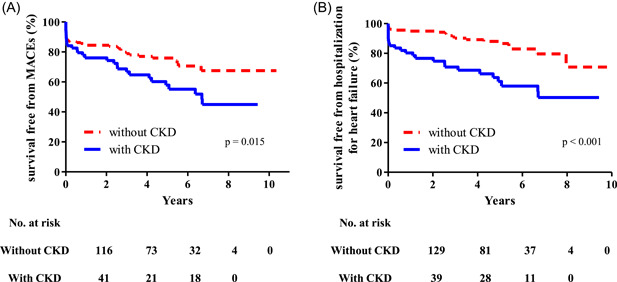
Kaplan–Meier curves for clinical outcomes in the overall study population. (A) Kaplan–Meier curves for major adverse cardiac events (MACEs) in the CKD group (solid line) and non‐CKD group (dashed line). (B) Kaplan–Meier curves for hospitalization due to heart failure. CKD, chronic kidney disease

Table 3 summarizes the rates of cumulative clinical outcomes and the risk of outcomes in both groups. The survival curves for primary and secondary outcomes using Kaplan–Meier analysis are presented in Figure 1. The median follow‐up duration was 41.5 (IQR: 16.2–71.7) months.

**Table 3:** Clinical outcomes compared between the groups with and without chronic kidney disease during follow‐up period

	Non‐CKD group (*N* = 192)	CKD group (*N* = 70)	*p* Value	Unadjusted HR (95% CI)	*p* Value	Adjusted HR (95% CI)	*p* Value
Composite outcome (MACEs)	43 (22.4%)	29 (41.4%)	.003	1.78 (1.11–2.85)	.017	1.71 (1.11–2.62)	.015
Hospitalization for heart failure	22 (11.5%)	26 (37.1%)	<.001	3.35 (1.90–5.93)	<.001	2.30 (1.16–4.55)	.017
Cardiac death	5 (2.6%)	7 (10.0%)	.018	1.78 (1.17–2.85)	.017	4.10 (1.02–16.41)	.046
Myocardial infarction	8 (4.2%)	3 (4.3%)	1.00	1.03 (0.27–3.89)	.96	0.56 (0.11–2.89)	.49
Aortic valve replacement	14 (7.3%)	2 (2.9%)	.25	0.35 (0.08–1.56)	.17	1.41 (0.26–7.64)	.69

*Note*: MACEs included cardiac death, myocardial infarction, hospitalization for heart failure, and aortic valve replacement.

Adjusted by age, sex, pulse pressure, hypertension, diabetes mellitus, hyperlipidemia, cerebrovascular accident, atrial fibrillation, and *e*ʹ velocity.

Abbreviations: CI, confidence interval; CKD, chronic kidney disease; HR, hazard ratio; MACEs, major adverse cardiac events.

Cox regression analysis revealed significant differences between the two groups in the rate of the primary outcome (MACEs: 41.4% vs. 22.4%, unadjusted HR: 1.78, 95% CI: 1.11–2.85, *p* = .017). The risk of MACEs in the CKD group was significantly different (adjusted HR: 1.71, 95% CI: 1.11–2.62, *p* = .015) on multivariate analysis using Cox regression model adjusted for age, sex, pulse pressure, HTN, DM, hyperlipidemia, cerebrovascular accident, atrial fibrillation, and *e*ʹ velocity.

Likewise, the risk of hospitalization for heart failure was significantly higher in the CKD group than in the non‐CKD group after adjustment (37.1% vs. 11.5%; adjusted HR: 2.30, 95% CI: 1.16‐4.55, *p* = .017). Additionally, the risk of cardiac death was also significantly higher in the CKD group (10.0% vs. 2.6%; adjusted HR: 4.10, 95% CI: 1.02–16.41, *p* = .046). However, there were no significant results in myocardial infarction and aortic valve replacement.

The results of the Cox regression analysis for primary and secondary outcomes are shown in Table 4. Multivariate Cox proportional hazard model with adjustment revealed that the presence of CKD was an independent prognostic factor for clinical outcomes (composite clinical outcome and heart failure for hospitalization).

**Table 4:** Univariate and multivariate Cox‐proportional analysis with adjustment for the prediction of clinical outcomes

	Univariate			Multivariate	*p* Value
	HR (95% CI)	*p* Value	*β*	Adjusted HR (95% CI)	
Composite outcome (MACEs)					
Age (years)	0.995 (0.974–1.016)	.64	−.024	1.005 (0.985–1.025)	.61
Sex (male)	1.168 (0.733–1.863)	.51	.120	1.017 (0.698–1.481)	.93
Chronic kidney disease (over Stage 3)	1.776 (1.107–2.848)	.017	.422	1.706 (1.111–2.620)	.015
Pulse pressure	1.019 (1.003–1.036)	.023	.020	1.007 (0.993–1.021)	.34
Hypertension	1.308 (0.599–2.859)	.5	.120	0.762 (0.437– 1.329)	.34
Diabetes mellitus	1.307 (0.750–2.279)	.35	.357	1.476 (0.930–2.340)	.10
Hyperlipidemia	1.131 (0.674–1.900)	.64	−.217	0.578 (0.369–0.905)	.017
Cerebrovascular accident	1.156 (0.645–2.073)	.63	.085	1.186 (0.731–1.925)	.49
Atrial fibrillation	1.496 (0.911–2.456)	.11	.391	0.995 (0.632–1.567)	.98
*e*ʹ Velocity (cm/s)	0.907 (0.820–1.002)	.054	−.103	0.904 (0.831–0.983)	.018
Hospitalization for heart failure					
Age (years)	1.048 (1.015–1.081)	.003	.015	1.015 (0.982–1.049)	.37
Sex (male)	1.369 (0.767–2.442)	.29	.358	1.430 (0.781–2.620)	.25
Chronic kidney disease (over Stage 3)	3.353 (1.896–5.930)	<.001	.833	2.299 (1.162–4.550)	.017
Pulse pressure	1.027 (1.006–1.048)	.011	.024	1.024 (1.001–1.047)	.041
Hypertension	1.897 (0.588–6.123)	.28	.197	1.217 (0.349–4.251)	.76
Diabetes mellitus	0.789 (0.369–1.687)	.54	−.147	0.863 (0.383–1.944)	.72
Hyperlipidemia	1.367 (0.695–2.688)	.37	−.180	0.836 (0.388–1.799)	.65
Cerebrovascular accident	1.753 (0.912–3.372)	.09	.224	1.252 (0.612–2.560)	.54
Atrial fibrillation	3.069 (1.738–5.418)	<.001	.648	1.912 (0.998–3.662)	.051
*e*ʹ Velocity (cm/s)	0.858 (0.762–0.966)	.012	−.065	0.937 (0.823–1.067)	.33
Cardiac death					
Age (years)	1.071 (1.001–1.146)	.046	.027	1.027 (0.961–1.098)	.43
Sex (male)	4.601 (1.008–21.002)	.049	1.504	4.500 (0.968–20.927)	.06
Chronic kidney disease (over Stage 3)	3.792 (1.200–11.976)	.023	1.410	4.096 (1.022–16.414)	.046
Pulse pressure	1.000 (0.959–1.043)	1.00	.006	1.006 (0.957–1.058)	.81
Hypertension	0.354 (0.095–1.329)	.12	−1.557	0.211 (0.039–1.148)	.07
Diabetes mellitus	0.376 (0.048–2.894)	.35	−.550	0.577 (0.066–5.051)	.62
Hyperlipidemia	0.411 (0.131–1.285)	.13	−.947	0.388 (0.089–1.684)	.21
Cerebrovascular accident	1.767 (0.478–6.533)	.39	−.052	0.949 (0.222–4.058)	.94
Atrial fibrillation	0.516 (1.432–14.237)	.010	1.310	3.708 (0.800–17.193)	.09
*e*ʹ Velocity (cm/s)	0.872 (0.686–1.108)	.26	−.027	0.974 (0.754–1.257)	.84
Myocardial infarction					
Age (years)	0.975 (0.931–1.020)	.27	−.029	0.971 (0.914–1.032)	.34
Sex (male)	0.514 (0.150–1.755)	.29	−.856	0.425 (0.111–1.620)	.21
Chronic kidney disease (over stage 3)	1.033 (0.274–3.893)	.96	−.580	0.560 (0.108–2.892)	.49
Pulse pressure	1.037 (0.995–1.081)	.08	.042	1.043 (1.000–1.087)	.049
Hypertension	1.619 (0.207–12.658)	.65	−1.084	0.338 (0.025–4.488)	.41
Diabetes mellitus	8.081 (2.365–27.619)	<.001	1.817	6.154 (1.659–22.832)	.007
Hyperlipidemia	5.231 (0.669–40.877)	.12	1.642	5.166 (0.430–62.088)	.20
Cerebrovascular accident	1.059 (0.229–4.902)	.94	−.030	0.970 (0.168–5.593)	.97
Atrial fibrillation	0.032 (0.000–10.778)	.25	−12.290	0.000 (0.000 ∼)	.97
*e*ʹ Velocity (cm/s)	0.952 (0.739–1.227)	.71	−.217	0.805 (0.566–1.145)	.23
Aortic valve replacement					
Age (years)	0.923 (0.890–0.958)	<.001	−.078	0.925 (0.887–0.964)	<.001
Sex (male)	2.060 (0.716–5.932)	.18	.559	1.748 (0.577–5.292)	.32
Chronic kidney disease (over Stage 3)	0.353 (0.080–1.559)	.17	.341	1.407 (0.259–7.643)	.69
Pulse pressure	0.979 (0.943–1.017)	.28	−.028	0.972 (0.934–1.012)	.17
Hypertension	0.952 (0.215–4.210)	.95	1.437	4.209 (0.775–22.870)	.10
Diabetes mellitus	0.602 (0.137–2.647)	.50	−.324	0.723 (0.149–3.505)	.69
Hyperlipidemia	0.411 (0.153–1.108)	.08	−1.238	0.290 (0.087–0.966)	.044
Cerebrovascular accident	0.322 (0.042–2.436)	.27	−.157	0.854 (0.100–7.333)	.89
Atrial fibrillation	0.206 (0.027–1.558)	.13	−1.250	0.287 (0.033–2.490)	.26
*e*ʹ Velocity (cm/s)	1.118 (0.907–1.378)	.29	−.017	0.983 (0.801–1.206)	.87

*Note*: MACEs included cardiac death, myocardial infarction, hospitalization for heart failure, and aortic valve replacement.

Adjusted by age, sex, pulse pressure, hypertension, diabetes mellitus, hyperlipidemia, cerebrovascular accident, atrial fibrillation, and *e*ʹ velocity.

Abbreviations: CI, confidence interval; HR, hazard ratio; MACEs, major adverse cardiac events.

## 4 DISCUSSION

This study evaluated the impact of CKD, a prognostic factor for hospitalization due to heart failure in patients with Stage B progressive AR. The principal findings were as follows: (1) the patients in the CKD group were significantly older and had higher pulse pressure and higher incidence of HTN, DM, dyslipidemia, cerebrovascular accident, and atrial fibrillation; (2) on multivariate Cox regression analyses, the risk of clinical outcomes (composite clinical outcome and hospitalization for heart failure) was significantly higher in the CKD group than in the non‐CKD group.

In the multivariate analysis about the risk of MACEs, CKD, hyperlipidemia, and *e*ʹ velocity were the statistically significant variables. And, in the multivariate analysis about risk of heart failure for hospitalization, CKD and pulse pressure were the statistically significant variables. In the study to identify prognostic factors, CKD, which proved statistical significance in predicting both composite clinical outcome and heart failure, would be the main result in this study.

Because of progressive dilatation and hypertrophy, the ventricle can maintain a normal stroke volume despite elevated afterload in the early phase of AR. In the literature, the mechanism underlying AR progression is explained as an increase in end‐systolic dimensions and ventricular wall stress leading to a gradual decline in LV function, resulting in the development of symptoms. Nevertheless, there are only a few published studies that have described the prognosis of mild and moderate AR.^13^, ^14^

Severe deterioration of LV function usually occurs insidiously, and the LV function may improve after correction of regurgitation.^1^, ^15^ Chronic severe AR brings in a state of gradual LV volume and pressure overload, resulting in progressive and eccentric hypertrophy and increment in LV dimensions to counter high wall stress, thus initially maintaining the low LV diastolic pressure. Even patients with severe chronic AR remain in this compensated and usually asymptomatic state for a very long time. Several studies have shown that subclinical LV myocardial dysfunction occurs early in the compensated stage when the LV ejection fraction is still preserved, that is, before the development of overt symptoms and often before reaching the current guideline‐recommended surgical thresholds.^16^, ^17^ A long asymptomatic period with adverse LV remodeling in response to persistent disease progression complicates and confuses the optimal timing of intervention. While estimating the outcomes, based on the progression of AR or occurrence of LV dysfunction, in patients with Stage B progressive AR, we should consider that the optimal timing of treatment may be too late. Therefore, hospitalization for heart failure might serve as an important endpoint for monitoring in these patients.

Patients with CKD suffer from a high burden of cardiovascular disease with increased cardiovascular mortality, an accelerated progression of atherosclerosis and valvular heart disease, an increased risk of congestive heart failure, and an increased risk of sudden cardiac death.^6^, ^8^, ^18^ Hence, patients with renal impairment frequently have a higher burden of severe morbidities, which may adversely affect long‐term survival.^19^ CKD might be the important metabolic cause associated with risk factors such as age and correlated with general calcification, degeneration, and inflammatory processes, as affecting the heart valve. Considering the gradual volume overload with respect to compensation in patients with AR, impaired renal function may affect the incidence of heart failure. This is because a proper volume control balance cannot be achieved. On adjusted‐multivariate analysis, we found that CKD was a factor of prognostic importance as it was associated with increased occurrence of adverse clinical events (composite clinical outcome and heart failure for hospitalization) in patients with AR.

In the literature, there are various findings on the impact of advanced CKD on aortic valve hemodynamics.^20^ The presence of volume load in a state of impaired kidney function contributes to cardiac chamber enlargement, which may worsen valvular regurgitation. Subsequently, the shear stress increases with progression in valvular disease. Moreover, the progression of valvular heart disease is faster in patients with CKD than in the general population. Furthermore, high volumes of back and forward flow in AR might affect renal blood flow in particular, thereby altering glomerular filtration pressure, leading to impaired kidney function. Although other studies have focused on higher cardiovascular mortality rates in aortic valve stenosis and posttranscatheter aortic valve intervention in patients with advanced CKD,^11^, ^21^, ^22^ there is limited data on cardiovascular mortality rates in other valvular abnormalities. This study verified a significant finding, that is, CKD contributes to clinical outcomes in patients with AR.

A shift in the age distribution of a population toward elderly individuals in the coming decades may further amplify the burden of AR on the healthcare system. Our results provide supporting evidence for developing echocardiography surveillance strategies endorsed by current guidelines and may assist in defining clinical recommendations for managing patients with Stage B progressive AR. Furthermore, given the prognostic role of CKD, the study results may help identify patients with increased risk of AR progression or excess risk of mortality requiring tailored therapy.

This study was a retrospective study, and all data were recorded in a single clinical database. We reported composite clinical outcomes.

Instead of a quantitative assessment, including regurgitant volume and regurgitant orifice area, an integrated echocardiographic assessment, with the final adjudication by a cardiac physiologist, was used to evaluate AR. We tried to evaluate the clinical outcome at a progressive stage rather than quantitative AR evaluation. Eventually, this could be a sufficient and well‐established methodology, as comprehensive quantitative assessment may not be feasible in all patients.

## 5 CONCLUSIONS

In the long term, impaired renal function negatively impacts the clinical outcomes (composite clinical outcome and hospitalization for heart failure) in patients with Stage B progressive AR. Although Stage B progressive AR should be carefully monitored in all patients, special attention should be paid to patients with pre‐existing CKD. Dedicated management efforts should be undertaken to optimize clinical adverse event (such as heart failure) prevention in these patients.

## CONFLICT OF INTERESTS

The authors declare that there are no conflict of interests.

## Data Availability

The data that support the findings of this study are available on request from the corresponding author.
